# Neural representation in F5: cross-decoding from observation to execution

**DOI:** 10.1186/1471-2202-16-S1-P190

**Published:** 2015-12-18

**Authors:** Murat Kirtay, Vassilis Papadourakis, Vassilis Raos, Erhan Oztop

**Affiliations:** 1 Computer Science, Ozyegin University, Istanbul, Turkey; 2 Foundation for Research & Technology- Hellas (FORTH) , Heraklion, Greece; 3 University of Crete Medical School, Heraklion, Greece

## 

Mirror neurons fire during both action execution and observation of a similar action performed by another individual [1]. However, this definition does not account for the existence of representational equivalence between execution and observation. To investigate this issue we recorded 68 neurons from area F5 of a macaque monkey trained either to execute reaching-to-grasp actions towards objects or to observe the experimenter performing the same actions [2], and adopted a decoding framework to find whether neurons effective in decoding the object/grip type in (1) execution and (2) observation conditions do exist, and most critically, to (3) assess whether transfer between execution and observation decoders (i.e. cross-decoding) can be employed. By 'transfer' we mean the application of the decoder parameters estimated using the neural discharge in observation to the neural firing recorded in execution, and vice versa. The success rate of such a decoder indicates the equivalence of representations in the two conditions.

Our analysis indicates that, at the level of single neurons, object/grip-specific decoders can be constructed, i.e. the type of the object/grip employed in either execution or observation can be decoded (success rate: 80%-100%, chance level: 25%). However, only in 10% of the cases (corresponding to the congruent type mirror neurons [1]) the decoder based on the execution discharge was effective when transferred to the observation discharge. The same was true for the reverse transfer. To extend this analysis at the population level we examined all pair performance of a 10-neuron set, consisted of 4 neurons having the best decoding performance and 6 neurons randomly selected. Out of the 45 possible pairs, 7 displayed high success rates (80% on average) in cross-decoding. Remarkably, high performing pairs were constituted only when one of the neurons displaying reliable decoding performance was paired with a randomly selected -poor solo decoder- neuron, which acted as a "helper". These results strongly point to a population based representation where good and poor decoders may cooperate to form a robust recognition system.

## Methods

Neuronal discharges during each condition were trimmed and represented as 14-bin histogram vectors. In the two neuron analysis, each neuron was reduced to a 7-bin histogram, and their concatenation results in a 14-tuple vector, to ensure similar decoder complexity (constant number of adjustable parameters). Thus, for each condition, ten 14-tuple neural firings (one per trial) made up the rows of the input matrix **X**, and the corresponding object ids (1-4) made up the output vector **Y**. We assumed a linear relation between input and output as **XW **= **Y**and solved for the weights (the decoder parameters) using the pseudo-inverse solution **W **= **X**^†^**Y**. Then, given a 14-tuple vector representation, **z**, of a discharge, the predicted object id is given by $y_{pred}=\underset{i=0..5}{\arg\min}\left\{\left[0,1,2,3,4,5\right]-z^{T}W\right\}$, where 0 and 5 indicates a definite wrong prediction. For execution-only and observation-only experiments, leave-one-out cross validation was applied to obtain the success rates in decoding. For cross-decoding analysis, the weight vector **W **obtained in one condition was used to predict the object type in the other condition by using the data from that condition.
